# Chemical Characterisation of Inorganic Profile of Wine Obtained by Alternative Vinification in Comparison with Traditional One

**DOI:** 10.3390/foods14111912

**Published:** 2025-05-28

**Authors:** Nicola Mercanti, Ylenia Pieracci, Monica Macaluso, Angela Zinnai, Olivier F. X. Donard, Véronique Vacchina

**Affiliations:** 1 Department of Agriculture, Food and Environment, University of Pisa, Via del Borghetto 80, 56124 Pisa, Italy; nicola.mercanti@phd.unipi.it (N.M.); monica.macaluso@unipi.it (M.M.); 2 Department of Pharmacy, University of Pisa, Via Bonanno 6, 56126 Pisa, Italy; yleniapieracci@gmail.com; 3 Interdepartmental Research Centre “Nutraceuticals and Food for Health”, University of Pisa, Via del Borghetto 80, 56124 Pisa, Italy; 4 Institut des Sciences Analytiques et de Physico-Chimie pour l’Environnement et les Matériaux CNRS, University of Pau & Pays Adour, UMR5254, Hélioparc Pau-Pyrénées, 2, Avenue du Président Angot, Cedex 9, 64053 Pau, France; olivier.donard@univ-pau.fr; 5 Ultra Traces Analyses Aquitaine (UT2A), Hélioparc Pau-Pyrénées, 2, Avenue du Président Angot, Cedex 9, 64053 Pau, France; veronique.vacchina@univ-pau.fr

**Keywords:** Sangiovese, ICP-MS analysis, vinification, sustainability, wine safety regulations

## Abstract

The complex dynamics between oxygen exposure, sulphur dioxide use, and wine quality are of the utmost importance in modern winemaking. While SO_2_ acts as an effective antiseptic and antioxidant, its excessive use raises health concerns, prompting stricter regulations (Council Regulation EC No. 1493/1999; Commission Regulation EC No. 1622/2000) and increasing interest in natural alternatives. In this context, Bioma SA developed plant-based additives derived from vineyard by-products rich in phenolic compounds to replace SO_2_ in vinification. This study has evaluated the impact of these additives on the inorganic elemental composition of Sangiovese wines, comparing traditional sulphite-based vinification with the Bioma-based alternative. Using triple quadrupole ICP-MS, 23 elements were quantified and analysed via ANOVA and principal component analysis (PCA). The results revealed significant effects of the vinification protocol and ageing method on key elements such as Mn, Rb, Sr, Ni, and As. Importantly, all toxic elements, Pb (≤5.9 µg/L), Cd (≤0.3 µg/L), and As (≤12.1 µg/L), remained well below EU safety thresholds. PCA further highlighted distinct elemental profiles between traditional and Bioma wines. These findings confirm that Bioma additives enable the production of wines with reduced sulphur content and compliant elemental safety, supporting their potential as sustainable, health-conscious alternatives in modern oenology.

## 1. Introduction

The study of wine’s chemical composition has become increasingly significant due to its potential to provide insights into wine’s origin, quality, and safety [1]. The inorganic profile of wine serves as a valuable indicator, reflecting both environmental and anthropogenic influences [2]. These elements, present in trace to macro concentrations, are shaped by factors such as soil composition, climatic conditions, and agricultural practices. Moreover, the elemental composition of wine plays a role not only in determining its sensorial properties but also in influencing its stability and preservation [3,4].

Several analytical approaches have been employed to characterise the elemental profile of wines, including flame atomic absorption spectrometry (FAAS), inductively coupled plasma optical emission spectrometry (ICP-OES), and, more recently, inductively coupled plasma mass spectrometry (ICP-MS), which provides high sensitivity and the ability to simultaneously detect trace, minor, and major elements [5,6,7]. Among these, triple quadrupole ICP-MS (ICP-QQQ) stands out for its ability to reduce spectral interferences and improve detection limits, making it a valuable tool for wine quality control and safety assessment [8].

Despite the growing literature on wine mineral content, there remains a lack of comprehensive studies that assess how innovative sulphite-free vinification techniques influence the elemental composition, particularly with regard to potentially toxic elements such as Pb, Cd, As, and Ni. These elements, even at trace levels, are regulated by European food safety authorities and must be carefully monitored in winemaking to ensure consumer protection [9,10]. This research deals with the application of a new analytical approach, based on the evaluation of multi-elemental composition, to differentiate wines obtained from the same grape variety (Sangiovese) but vinified using two different methods: one vinification followed a traditional protocol with the addition of potassium metabisulphite, while the other one followed an alternative protocol in which alternative natural additives were used, as described in previous work [11]. The study aims to determine whether the use of natural additives as alternatives to sulphur dioxide results in observable differences in the wine elemental fingerprint. The essential inorganic elements typically present in wine include Ca, Co, Cu, Fe, K, Mg, Mn, Mo, Na, and Zn. These metals are usually found as ions or complexes [12], and the majority of them, like Co, Cu, Fe, Mn, Mo, Ni, and Zn, are considered essential for healthy plant growth [13] and show minimal effects on sensorial characteristics of wine compared to other components. In addition to beneficial metals, wine also contains non-essential metals and metalloids such as Al, As, Cd, Cr, Ni, and Pb, which can exist in various oxidative states. These elements are typically present in trace quantities in wine and do not offer any positive effects on the body. However, if present in high concentrations, they may become toxic [14].

Inorganic element content is therefore determined by a wide range of factors, such as climate, soil, topography, and cultivation/harvesting method [15], as well as by anthropogenic activity. Indeed, it is possible to discriminate between different elements and associate them with their different origins. For example, while Al, B, Ba, Li, Mn, Mo, Rb, and Sr are not influenced by production processes [16], other elements can be influenced by both natural and anthropic factors. Among them, Ca and Mg are natural constituents of the musts, even though they are also derived from the addition of carbonates as disacidificant [17]. While Cu and Zn come naturally from the soil, they are also contained in fungicidal treatments or vinification equipment [18]. Fe is naturally present in the soil, but it can also be derived from artificial sources such as vinification equipment and steel containers [19]. Further, K is the predominant macro-element of the wine, but it can also be added as metabisulphite or carbonate during vinification. Analogously, S can be derived from chemical treatments with metabisulphite [20]. Na can be derived from the soil, but it also can be added illicitly in salting treatments, particularly during the winemaking process, to improve the stability and preservation of wine. *p* is present naturally as both organic and inorganic phosphates, but it can be added as calcium or ammonium salts.

Finally, there are elements almost completely influenced by artificial sources, such as Pb (fungicidal treatments, vehicular traffic), Co, Cr, Ni, and V (metallic containers), which are usually present in wines in very low concentrations [21].

In this study, 23 elements were selected on the basis of literature data and representing markers of soil, agricultural practices, and anthropogenic contamination to investigate the elemental fingerprint of the samples analysed in the present study. They were classified as major or minor based on their typical concentration ranges in wine matrices. Major elements (macroelements), such as potassium (K), calcium (Ca), magnesium (Mg), sodium (Na), and sulphur (S), are usually present at levels ranging from tens to thousands of mg/L or even g/L. Minor or trace elements, including metals such as iron (Fe), copper (Cu), manganese (Mn), and zinc (Zn), as well as toxic elements like lead (Pb), cadmium (Cd), arsenic (As), and nickel (Ni), occur at much lower concentrations, typically in the µg/L range. All the elements were analysed in detail by a triple quadrupole ICP-MS, which allows us to analyse all of the elements at the same time, as it is able to eliminate interferences.

## 2. Materials and Methods

### 2.1. Wine Samples

Wine samples, both Bioma and Traditional, whose vinification protocol has been explained previously [11], were collected at the end of the ageing phase before bottling and analysed to evaluate their elemental fingerprint. Both the vinification procedures were obtained starting from musts with the same characteristics.

### 2.2. Reagents and Materials

Nitric acid 69.0–70.0%, Instra Analysed Reagent, Fisher Scientific Bioblock (Illkirch, France). H_2_O_2_ 30% (Fisher Scientific Bioblock, Illkirch, France).

Mono-elemental stock standards containing 1000 µg/mL (10,000 µg/mL in the case of K) were purchased from CPAchem (Trappes, France). All working standard solutions were prepared daily in ultrapure water. (18 MΩ/cm) (Millipore, Bedford, MA, USA).

### 2.3. Sample Mineralisation

1 mL of each wine sample was subjected to mineralisation with the addition of 1 mL of HNO_3_. Once the reaction was finished, 500 µL of H_2_O_2_ was added, and the resulting solution was digested using a Hotblock Digestion System DigiPREP (SCP Sciences, Courtaboeuf, France) for 2 h at 85 °C. After cooling, the solution was diluted with ultra-pure water up to 50 mL. Three blank samples were prepared in the same way as the studied samples.

### 2.4. ICP-MS Measurement Conditions

The quantification of the 23 elements was performed by means of a triple quadruple ICP-MS (Thermo Fischer, iCAP TQe, Waltham, MA, USA). The ICP-MS measurement conditions were optimised daily to give the highest intensity using a standard built-in software procedure.

The ICP-MS settings are described in Table 1 for every element investigated.

The use of the ICP-MS in the SQ-KED mode with a dwell time of 0.1 s and a normal resolution was the default mode used, particularly for trace elements. To deal with some potential polyatomic interferences, the ICP-MS was used in the TQ-O2 mode, either in the mass-shift (the *m*/*z* of the element of interest is shifted after reaction with O_2_ whereas the *m*/*z* of the interferent is not changed) or on-mass (the *m*/*z* of the interferent is shifted without impacting the *m*/*z* of the element of interest) mode. For major elements (K, Mg, Na…), in order to protect the detector of the ICP-MS, the intensity of the signal was artificially lowered. For this, the dwell time was decreased by at least a factor of 10 and the quadrupoles were used in the high resolution mode to reduce the bandwidth and, therefore, the amount of ions sent to the detector. This allows us to lower the intensity of the signal measured and, therefore, the sensitivity of the measurement without significantly impacting the accuracy of the measurement. However, this was applied only for major elements for which sensitivity is not an issue.

### 2.5. Statistical Analyses

One-way analysis of variance (ANOVA) was performed to evaluate statistical differences in the element concentrations among the different samples. Replicate wine samples were independently digested, and each element was quantified in six replicates (n = 6) per condition.

Averages were separated by Tukey’s post-hoc test and using a *p* < 0.05.

All data are expressed as mean ± standard deviation (SD).

Two-way ANOVA was further performed to evaluate the influence of winemaking protocol, ageing method, and their interaction on the elemental fingerprint. The presence of significant influence was assessed by Tukey’s post-hoc test and using a *p* < 0.05. Principal component analysis (PCA) methods through employing the JMP Pro statistical package 17 (SAS Institute; Cary, NC, USA) were also applied to visualise group separation.

## 3. Results and Discussions

The multi-elemental fingerprint of the analysed samples is reported in Table 2. Results evidenced the presence of great concentrations of elements like Mg, Mn, Ca, K, and S, while Cd, Pb, Co, Cr, Ni, and V, according to the literature, were found in low concentrations [20]. Sodium levels in wine were influenced by both winemaking and ageing methods (Table 3). Sodium can originate from the soil or be introduced through the addition of sodium metabisulphite as a preservative [16,22,23]. Excessive sodium can alter the taste of wine, making it less desirable [24]. Potassium is the predominant macro-element in wine, and its levels were consistent across different winemaking and ageing methods (Table 3). Potassium is crucial for the stability of wine, particularly in regulating its pH, and is often added during the winemaking process in the form of potassium metabisulphite [25,26].

Manganese is essential for plant growth and was one of the most abundant elements in the samples [27,28]. The study evidence significant differences in manganese levels based on winemaking and ageing methods (Table 3). Magnesium is essential for plant health [29] and was one of the more abundant elements in the samples (Table 2), with minimal differences observed between the different methods. Magnesium plays a key role in chlorophyll production and grape metabolism, contributing to the overall quality of the win [30]. Sulphur, commonly introduced in wine through the use of sulphur dioxide as a preservative [31], showed high variation across samples (Table 2). Sulphur is critical for wine stability and preservation, particularly in preventing oxidation and microbial growth [32]. Calcium levels, like those of Mg, were relatively uniform across the different samples (Table 2). Calcium is important in stabilising tartaric acid in wine, and its presence is influenced by both natural sources and winemaking practices [33].

All the major elements, with the exception of manganese, did not show significant differences among the samples, as evidenced by ANOVA analysis. In detail, manganese seemed to be influenced by both the winemaking protocol and the ageing method, as demonstrated by the two-way ANOVA analyses (Table 3), and these findings did not corroborate with the literature. In addition to manganese, two-way ANOVA also evidences the influence of the same factors on the content of rubidium, strontium, nickel, and arsenic. Rubidium levels were significantly affected by both winemaking and ageing (Table 3), with wood-aged wines showing higher concentrations (9.9 ± 0.58 mg/L for Bioma aged in wood and 9.2 ± 0.2 µg/L for traditional wood as reported in Table 2). Rubidium is not essential for human health but can act as a geographic marker, helping to distinguish wines from different regions or production methods. Strontium, like rubidium, is commonly used as a geographical indicator in wine studies [34]. Results showed that strontium levels were affected by the winemaking protocol (Table 3), with Bioma wines displaying higher concentrations, as evidenced in Table 2. Its presence, typically from the soil, may be helpful in tracing the origin of the used grapes. Nickel concentrations varied significantly across the samples (from 22.3 ± 0.3 to 34.3 ± 0.3 µg/L) and are influenced by both winemaking techniques and ageing. Though its concentrations were low (Table 2), monitoring nickel is important, as excessive levels can lead to toxicity.

Arsenic is a toxic element [10], and its concentration in wine was found to be influenced by both winemaking and ageing methods (Table 3). Although present in low concentrations, arsenic requires strict monitoring due to its potential health risks.

Regarding the other elements commonly found in wine, the only two that seemed mostly influenced by the interaction between ageing and winemaking techniques were copper and iron (along with Al, Ni, Pb, V, Cr, Pb, and Na), as shown in Table 3.

The present study evidenced that copper concentration was influenced by the ageing method, resulting in higher levels in wood-aged wines (Table 2). In addition, the interaction between the ageing technique and winemaking method significantly influenced copper levels (Table 3). This element usually comes from the soil, but they are also contained in fungicidal treatments or vinification equipment. Understanding its origin may be helpful for controlling its level in wines, as it is responsible for oxidation issues. Concerning the other elements, Barium and Molybdenum, contrary to the literature, were influenced by winemaking protocol (Table 3), while boron and lithium seemed to be not influenced by different types of production, as confirmed by the literature [16].

Lithium showed significant differences among the samples, and their concentration was influenced by the winemaking protocol but not by the ageing method. This element, though present in trace amounts, primarily derives from the soil and can act as a potential geographic marker of the vineyard. The variation observed suggests that lithium may be used to trace differences between Bioma and a traditional one.

Boron, which plays a vital role in plant growth [14], was found in relatively consistent quantities across most samples, suggesting less influence on the winemaking or ageing processes.

Chromium concentration was significantly influenced by the ageing method, particularly in wood-aged wines. The higher concentrations of chromium observed in wines aged in steel vessels are likely attributable to metal leaching from the winemaking equipment. Steel commonly used in oenology contains Chromium, which can be released under acidic conditions typical of wine, especially in the presence of organic acids such as tartaric and malic acid [35,36,37]. Additionally, oxidative conditions and long contact times may enhance this release [38]. In contrast, wood barrels do not contribute this metal to the wine and may even act as passive adsorbents through interactions with polyphenolic compounds, thus reducing the concentration [39,40].

Zinc content was influenced by both winemaking protocol and ageing methods (Table 3). High concentrations of zinc may result from the use of zinc-containing fungicides. It is important to maintain optimal levels of this element, as an excessive concentration can lead to toxicity in both plants and humans [41,42]. For this reason, the monitoring of this element is of the utmost importance for consumer protection.

Molybdenum was found in low concentrations across all samples (Table 2). The study indicates that the concentration of this element was more influenced by the winemaking protocol than by the ageing process (Table 3). Its low concentration in wine is generally related to minimal health risks, but even in low amounts, it represents a key element for grapevine health [43].

Cadmium is a toxic metal, and its presence in wine is undesirable [10]. The study revealed that cadmium levels varied significantly with both winemaking and ageing processes (Table 3). Barium concentration, like cadmium, was influenced by both winemaking techniques and ageing, resulting in higher levels in wood-aged wines (78.1 ± 1.0 µg/L for Bioma and 62.3 ± 0.8 µg/L for Traditional). Lead is another toxic element, and its concentration in wine must be carefully regulated [10]. The study found significant differences in lead levels, with wood-aged wines showing higher concentrations (Table 2). Lead is often associated with anthropogenic sources such as vehicular traffic or fungicides. Monitoring lead levels is crucial to ensure wine safety, as elevated levels can pose serious health risks [44].

Aluminum was found in trace amounts, and its presence was influenced by winemaking equipment, such as aluminium containers. Though not essential, Aluminum is typically not a health concern in the low concentrations found in wine, but excessive exposure should still be avoided [45].

Vanadium levels were affected by both winemaking techniques and ageing methods (Table 3), with wood-aged wines generally showing higher levels (Table 2). Finally, copper concentration, although found in low amounts, was influenced by both the winemaking protocol and the ageing method, as was the case with vanadium content. Its presence is usually attributed to contamination from industrial or agricultural sources.

However, lead, cadmium, and arsenic were present below EU limits (Pb: ≤5.9 µg/L; Cd: ≤0.3 µg/L; As: ≤12.1 µg/L), confirming regulatory compliance and consumer safety. These concentrations are in line with the maximum levels set by Commission Regulation (EC) No 1881/2006, which sets limits for contaminants in food, including lead and cadmium in wine, and Regulation (EU) 2015/1005 amending Directive 98/83/EC on the quality of water intended for human consumption as regards nickel and arsenic. These levels are well below the maximum permissible concentrations set by EU legislation for wine safety. (Pb: ≤5.9 µg/L; Cd: ≤0.3 µg/L; As: ≤12.1 µg/L), indicating full adherence to EU safety limits and consumer safety [46]. These levels are well below the maximum concentrations permitted by European legislation for wine safety. These results support and build upon the preliminary investigations conducted in a previous work [11], which initially demonstrated the viability of using natural additives derived from the vineyard as sulphur dioxide substitutes in winemaking. While that focused primarily on the aromatic and chemical composition of wines, this research provides a complementary perspective by clarifying the inorganic elemental footprint associated with such alternative practices. The detailed multi-elemental profile and statistical analysis presented here offer new evidence that these additives not only maintain aromatic and chemical quality but also influence the concentration and variability of specific elements (e.g., Mn, Sr, Rb, Ni and As). These compositional differences can serve as both safety indicators and potential markers of authenticity, reinforcing the broader applicability of the sulphite-free approach introduced in previous research [7].

**Figure 1:**
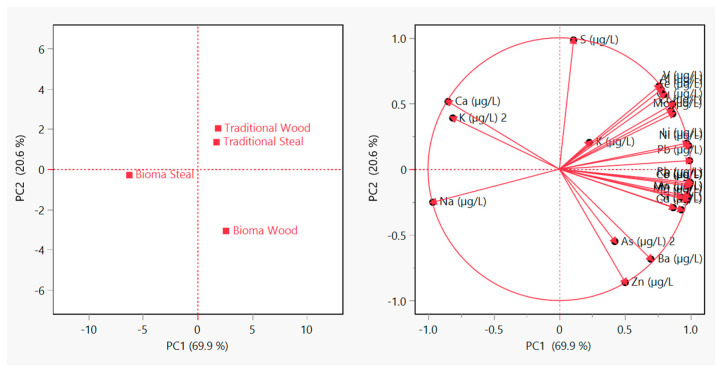
Principal component analysis (PCA) of the elemental composition of Sangiovese wines obtained by traditional and Bioma vinification protocols, aged in wood and steel.

Figure 1 shows the results of the principal component analysis (PCA) performed on the concentrations of 23 elements detected in the wine samples. The first two principal components (PC1 and PC2) explain 69.9% and 20.6% of the total variance, respectively, accounting for 90.5% of the overall dataset variability. The score plot (left panel) reveals a clear separation between wines produced using Bioma and traditional protocols, with Bioma Wood samples showing the most distinct profile along PC1. This indicates that the alternative vinification significantly influences the inorganic elemental composition of the final product, particularly when combined with wood ageing. The loading plot (right panel) highlights that elements such as manganese, rubidium, strontium, arsenic, and zinc contribute most strongly to the separation along PC1, while Sodium, Calcium, and Potassium exhibit inverse correlations. These findings confirm that elemental profiling can effectively discriminate between vinification protocols and support the traceability potential of the Bioma-based method.

## 4. Conclusions

This study successfully highlighted the influence of different winemaking techniques and ageing methods on the elemental composition of Sangiovese wine. Results demonstrate that while certain essential elements like Mg, Ca, K, and S show little variation across the different samples, elements such as Mn, Cu, Fe, and others are significantly impacted by both winemaking protocols and ageing methods. Additionally, some non-essential and potentially toxic elements (e.g., Cd, Pb, Ni) also vary in concentration, which highlights the need for careful monitoring. In line with previously results [11], this study confirms that alternative winemaking using natural additives can achieve results comparable to those of traditional sulphite-based methods. By incorporating multi-elemental fingerprinting into wine quality assessment, our research strengthens the scientific basis for the adoption of sulphite-free winemaking approaches and underlines the potential of elemental profiling as a tool for quality assurance and traceability. Future research should expand the dataset to include multiple vintages, grape varieties, and terroirs and aim to correlate elemental profiles with organoleptic properties and shelf life. Such data would reinforce the role of elemental fingerprinting not only as a tool for traceability but also for predicting quality, safety, and market potential.

## Figures and Tables

**Table 1:** Operating parameter values used for ICP-QQQ-MS.

Elements	Mode	Dwell Time	Resolution
Ni, Cu, Zn, Rb, Sr, Mo, Cd, Pb, Mn, Co	SQ-KED	0.1 s	Normal
V, As, Ba	TQ-O2 (mass-shift mode)	0.1 s	Normal
S, Ca, Cr		0.01 s	High
Al, Li, B	TQ-O2 (on-mass mode)	0.1 s	Normal
Mg, Na		0.01 s	High
K, Fe		0.005 s	High

**Table 2:** Elemental composition of the analysed wines.

**Samples**	**Ca (mg/L)**	**Mg (mg/L)**	**K (g/L)**	**Na (mg/L)**	**S (mg/L)**	**Al (µg/L)**	**As (µg/L)**	**Ba (µg/L)**	**Br (mg/L)**	**Cd (µg/L)**	**Co (µg/L)**	**Cr (µg/L)**	**Cu (µg/L)**
Bioma aged in wood	280 ± 151 ^A^	536 ± 273 ^A^	1.92 ± 0.09 ^A^	10.0 ± 0.2 ^B^	107 ± 4.67 ^A^	567 ± 41 ^BC^	12.1 ± 0.5 ^A^	78.1 ± 1.0 ^A^	10.7 ± 0.04 ^A^	0.3 ± 0.1 ^A^	5.7 ± 0.2 ^A^	20.5 ± 0.4 ^BC^	186 ± 1 ^B^
Traditional Wood	407 ± 160 ^A^	474 ± 318 ^A^	1.98 ± 0.17 ^A^	7.2 ± 0.01 ^B^	209 ± 11 ^A^	721 ± 25 ^AB^	11.1 ± 0.1 ^B^	62.3 ± 0.8 ^BC^	10.1 ± 0.1 ^AB^	0.3 ± 0.1 ^A^	5.3 ± 0.2 ^B^	24.3 ± 0.8 ^A^	248 ± 4 ^A^
Traditional Steel	367 ± 231 ^A^	454 ± 168 ^A^	1.87 ± 0.11 ^A^	6.12 ± 0.2 ^A^	180 ± 86 ^A^	649 ± 11 ^C^	11 ± 0.0 ^C^	58.5 ± 0.8 ^C^	10.0 ± 7 ^B^	0.2 ± 0.1 ^B^	4.9 ± 0.2 ^C^	28.0 ± 2.0 ^C^	255 ± 1.1 ^C^
Bioma Steel	494 ± 235 ^A^	295 ± 164 ^A^	1.90 ± 0.20 ^A^	26.01 ± 0.8 ^B^	150 ± 55 ^A^	465 ± 17 ^A^	8.5 ± 0.1 ^AB^	52.6 ± 0.6 ^B^	7.02 ± 0.04 ^AB^	0.2 ± 0.1 ^B^	3.0 ± 0.2 ^AB^	12.5 ± 0.7 ^AB^	99 ± 3 ^AB^
**Samples**	**Fe (mg/L)**		**Li (µg/L)**	**Mn (mg/L)**	**Mo (µg/L)**	**Ni (µg/L)**	**Pb (µg/L)**	**Rb (mg/L)**	**Sr (µg/L)**		**V (µg/L)**	**Zn (µg/L)**	
Bioma aged in wood	3.2 ± 0.1 ^BC^		10.8 ± 0.4 ^A^	1.86 ± 0.7 ^A^	2.2 ± 0.1 ^C^	34.3 ± 0.3 ^A^	5.8 ± 0.1 ^A^	9.90.58 ^A^	697 ± 6 ^A^		2.0 ± 0.1 ^B^	837 ± 9 ^A^	
Traditional Wood	4.6 ± 1.0 ^A^		11.0 ± 0.4 ^A^	1.78 ± 0.6 ^B^	2.4 ± 0.1 ^A^	35.7 ± 0.5 ^A^	5.9 ± 0.1 ^A^	9.2 ± 0.2 ^A^	670 ± 8 ^B^		2.6 ± 0.1 ^A^	630 ± 9 ^B^	
Traditional Steel	5 ± 0 ^C^		11.3 ± 0.3 ^A^	1.60 ± 0.6 ^C^	2.6 ± 0.1 ^A^	35.4 ± 0.1 ^B^	5.5 ± 0.1 ^B^	9.0 ± 0.0 ^B^	612 ± 6 ^C^		2.7 ± 0.1 ^B^	639 ± 10 ^B^	
Bioma Steel	2.6 ± 0 ^AB^		8.5 ± 0.4 ^B^	1.38 ± 0.6 ^A^	1.8 ± 0.1 ^B^	22.3 ± 0.3 ^A^	3.5 ± 0.1 ^A^	5.58 ± 0.01 ^A^	554.7 ± 11.6 ^A^		1.6 ± 0.1 ^A^	613 ± 3 ^B^	

Superscript uppercases (A, B, C) indicate statistical differences among the samples evaluated by Tukey’s post-hoc test, with *p* < 0.05.

**Table 3:** Two-way ANOVA was performed to evaluate the influence of the winemaking protocol.

Element	Winemaking Protocol (WP)	Ageing Method (AG)	WP × AG
**Ca (mg/L)**	*	n.s.	*
**Mg (mg/L)**	n.s.	n.s.	*
**K (g/L)**	n.s	n.s.	n.s.
**Na (mg/L)**	**	**	***
**S (mg/L)**	n.s.	n.s.	*
**Al (µg/L)**	*	n.s.	***
**As (µg/L)**	***	***	**
**Ba (µg/L))**	***	***	*
**Br (mg/L)**	**	n.s.	n.s.
**Cd (µg/L)**	***	**	*
**Co (µg/L)**	***	***	**
**Cr (µg/L)**	n.s.	**	*
**Cu (µg/L)**	n.s.	*	***
**Fe (mg/L)**	n.s.	*	***
**Li (µg/L)**	**	n.s.	**
**Mn (mg/L)**	n.s.	*	n.s.
**Mo (µg/L)**	***	**	*
**Ni (µg/L)**	**	**	***
**Pb (µg/L)**	***	**	***
**Rb (mg/L)**	***	***	**
**Sr (µg/L)**	***	***	*
**V (µg/L)**	n.s.	n.s.	***
**Zn (µg/L)**	***	***	**

* *p* < 0.05; ** *p* < 0.01; *** *p* < 0.0001; n.s. not significative.

## Data Availability

The original contributions presented in the study are included in the article; further inquiries can be directed to the corresponding author.
